# Quality of Life and Wellbeing in Peripheral Arterial Disease: A Qualitative Study

**DOI:** 10.1093/geroni/igab046.3729

**Published:** 2021-12-17

**Authors:** Mary Byrnes, Heather Dillaway, Bryan Aaron, Alisha Heximer, Valeria Valbuena, Matthew Corriere, Nicholas Osborne

**Affiliations:** 1 University of Michigan, Ann Arbor, Michigan, United States; 2 Wayne State University, Wayne State University, Michigan, United States; 3 University of Michigan Medical School, University of Michigan Medical School, Michigan, United States; 4 University of Michigan, University of Michigan Medical School, Michigan, United States; 5 University of MIchigan, University of Michigan, Michigan, United States; 6 University of Michigan, University of Michigan, Michigan, United States

## Abstract

Peripheral arterial disease (PAD) is a vascular condition disproportionately affecting adults > 60 and the leading cause of disability for adults > 50. Because PAD is marked by severe leg pain and sometimes lower extremity amputation, quality of life (QOL) and wellbeing may be compromised however, we understand little about these constructs in this population. Furthermore, surgical care providers lack a comprehensive understanding of how individuals think about wellbeing and what is important to individuals during surgical care. We conducted a qualitative photographic elicitation study (n = 60) in one academic multidisciplinary PAD clinic to understand specific aspects of QOL of older individuals with PAD. Guided by interpretive description, a methodology pioneered in nursing, we analyzed data within and across five clinical symptom severity categories to examine for QOL constructs, impact on everyday life, understanding of disease, and desired treatment. Results demonstrate that individuals do not fully understand PAD diagnosis or its implications (e.g., “[I] have never heard of it. Do I have that?”). Disease-specific knowledge was prevalent among patients experiencing lower extremity amputation but those suffering from wounds or gangrene had limited understanding. Furthermore, patients’ descriptions of QOL conflicted with the conceptualization of QOL in clinical practice and research. That is, many participants describe QOL based on activities they are capable of performing despite limitations. Results demonstrate the need for integrating gerontological knowledge into clinical care to improve quality of care for older adults.

